# Biosynthesis approach of copper nanoparticles, physicochemical characterization, cefixime wastewater treatment, and antibacterial activities

**DOI:** 10.1186/s13065-023-00982-7

**Published:** 2023-07-09

**Authors:** Esraa Hassan, Ahmed A. Gahlan, Gamal A. Gouda

**Affiliations:** Department of Chemistry, Faculty of Science, Al-Azhar University, Assiut Branch, Assiut, 71524 Egypt

**Keywords:** Copper, Nanoparticles, Cefixime, Wastewater, Antibacterial

## Abstract

The aim of this paper is the green synthesis of copper nanoparticles (*Cu NPs*) via* Quinoa* seed extract. X-ray diffraction (XRD) results confirmed the production of the pure crystalline face center cubic system of the *Cu NPs* with an average crystallite size of 8.41 nm. Infrared spectroscopy (FT-IR) analysis confirmed the capping and stabilization of the *Cu NPs* bioreduction process. UV visible spectroscopy (UV–Vis). surface plasmon resonance revealed the absorption peak at 324 nm with an energy bandgap of 3.47 eV. Electrical conductivity was conducted assuring the semiconductor nature of the biosynthesized *Cu NPs*. Morphological analysis was investigated confirming the nano-characteristic properties of the Cu NPs as polycrystalline cubic agglomerated shapes in scanning electron microscopy (SEM) analysis. Transmission electron microscopy (TEM) analysis also was used to assess the cubic shapes at a particle size of 15.1 ± 8.3 nm and a crystallinity index about equal to 2.0. Energy dispersive spectroscopy (EDX) was conducted to investigate the elemental composition of the *Cu NPs*. As a potential utility of the biosynthesized *Cu NPs* as nano adsorbents to the removal of the Cefixime (*Xim*) from the pharmaceutical wastewater; adsorption studies and process parameters were being investigated. The following strategic methodology for maximum Xim removal was conducted to be solution pH 4, Cu NPs dosage 30 mg, Xim concentration 100 mg/L, and absolute temperature 313 K. The maximum monolayer adsorption capacity was 122.9 mg/g according to the Langmuir isothermal model, and the kinetic mechanism was pseudo-second-order. Thermodynamic parameters also were derived as spontaneous chemisorption endothermic processes. Antibacterial activity of the *Xim* and *Xim@Cu NPs* was investigated confirming they are highly potent against each Gram-negative and Gram-positive bacterium.

## Introduction

The necessity to disinfect wastewater is critical to the globe today. There are several ways for water to become polluted. Contamination from pigments, chemicals, and pharmaceutical wastewater is one of these causes [1, 2]. Water containing organic contaminants, such as pharmaceutical waste and chemicals produced by textile industries, has an effect on the natural process, namely the photosynthesis process in plants, which has a direct influence on aquatic species and an indirect effect on human life. Contaminated water also has a major impact on the lives of animals, birds, and people who are affected by contaminated flowing water. [3]. Numerous research studies have found that water pollutants such as pharmaceutical residues and colors can cause disease, infertility, and mutations [4–6].

Resistance to antibiotics has been established as one of the most severe concerns affecting global health in the twentieth century. Drug resistance has reached pandemic proportions around the world, particularly in developing countries. Previous research showed antibiotic-resistant bacteria and antibiotic-resistance genes in wastewater [7, 8]. Despite their low concentrations, the antibiotics proved hazardous to aquatic life. Many scientists are interested in studies on the presence and destiny of antibiotics, as well as the removal of antibiotics from aqueous solutions. Many physicochemical techniques and technologies, such as degradation through the photocatalytic process [9–11], membrane processes [12], advanced oxidation [13], ozonation process [14], and adsorption technique, can be utilized to remove antibiotics from pharmaceutical industry effluent and waste [15–23].

The most common oral antibiotics are macrolides and cephalosporin, which are found in large quantities in pharmaceutical wastewater from the factories of these antibiotics. Ceftriaxone and Cefixime* (Xim)* are third-generation antibiotic cephalosporin members and are examples of the most consumable cephalosporin antibiotics [24, 25]. *Xim* is semi-synthetically synthesized from the *Cephalosporium acremonium*, a marine fungus. It can be used for treatments of numerous and different bacterial infections and it has excellent activity against several pathogens such as *Anaerobes*, *Enterobacteriaceae* Gram-negative classes such as *Branhamella catarrhalis*, *Escherichia* *coli*, *Neisseria gonorrhoeae*, *Klebsiella*, *Haemophilus* influenza, *Serratia marcescens*, *Providencia*, *Haemophilus,* and *Meningococcus*. Because it has a safety profile, it considers the optimal oral antibiotic for switch therapy, according to its high efficacy. Also, it has high quality; *Xim* is used to kill bacterial growth [25].

Metal and metal oxide nanoparticles are mostly used for water remediation and purification. Among the transitional elements, copper could be chosen to prepare nanoparticles because it has several factors, the most important of which is its relative abundance. Its salts are cheap compared to silver and gold salts [26, 27]. It is involved in many biological activities of the human body [28]. It also has many applications that have a wide scope as a potential application, where recent studies have proven the possibility of using it as an antibacterial of both types Gram-positive and Gram-negative, against some types of cancerous tumors [29–32]. Also, some important and smart applications were hinted at as wound healing activity [33, 34]; anti-inflammatory [20], and water purification [35, 36].

Green synthesis has several benefits in nanoscience and technology. The use of environmentally friendly raw materials and non-toxic diluters is critical in the production and processing of nanoparticles, necessitating the employment of environmentally acceptable techniques [37–39]. Many different parts of several plants could be used for the green bio-fabrication of *Cu NPs* [40, 41]. *Quinoa* is a plant that follows the *Chenopodiaceae* family. Phytoconstituent studies of *Quinoa* seeds revealed the presence of multi constituents as flavonoids, protein as essential amino acids (11–19%), carbohydrates (starch about 52–69%), lipids as sulfur amino acids and lysine (15%), vitamins as pyridoxine (B6) and folic acid, ascorbic acid, vitamin E [42].

**Fig. 1:**
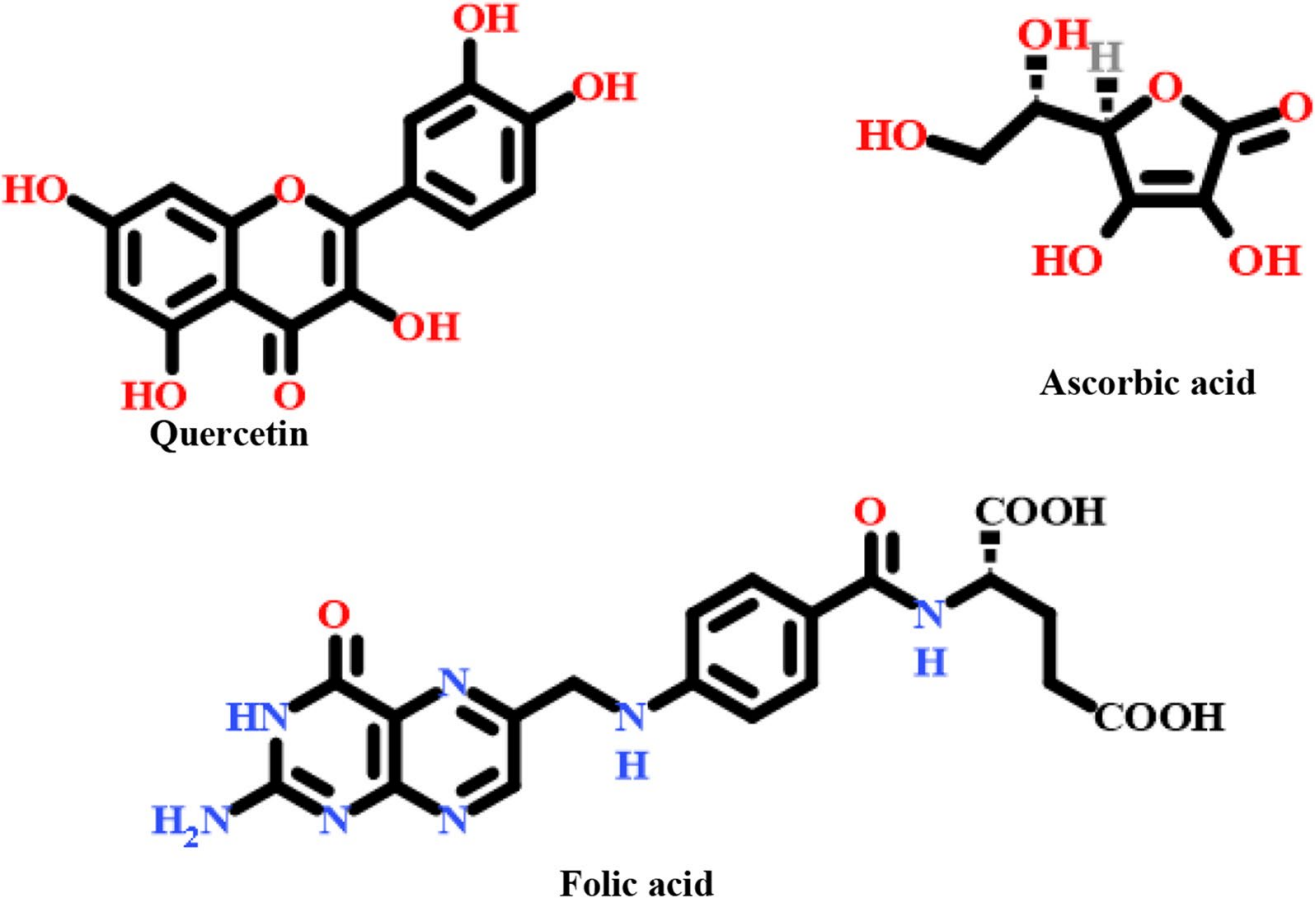
Some of the investigated bioactive molecules are present in the *Quinoa* seeds

Additionally, *Quinoa* seeds have biological activities that were be revealed based on the polyphenols contents that recently conducted in different investigations [43]. Also, *Quinoa* extracts a rich source of antioxidants for the improvement of other functions of cells in various fields of medicine according to its contents such as sinapinic, ferulic, gallic acids, isorhamnetin, kaempferol, and rutin [44–46]. (Fig. 1) shows the structure for some of these Phyto molecules that act as a reducing agent that could be used for copper ions bioreduction.

The current research employs Quinoa seed extract for the first time to achieve the green biosynthesis of Cu NPs. Following that, physicochemical characterization was carried out utilizing several techniques to corroborate the biofabrication of Cu NPs at the nanoscale. Following that, the removal remediation of cefixime antibiotic by adsorption approach was explored by analyzing the various influencing parameters of the new generation nano-adsorbents. The use of *Cu NPs* in the adsorption process considers one of the most practical applications to utilize the high surface area of the biosynthesized nanoparticles. The essential impact of parameters such as solution pH, adsorbent dose, adsorbate concentration, temperature change, and contact time was conducted. Isotherms, kinetics, and thermodynamics were conducted to understand the behavior of the *cefixime* adsorption process onto the *Cu NPs* surfaces. Also, *Cu NPs* and Xim@*Cu NPs* antibacterial activities were studied against different species of Gram-positive and negative bacteria.

## Experimental

### Materials and reagents

All chemical materials used in the present work were analytical grade. Methanol HPLC grade (Merck, UK). Copper sulfate pentahydrate [CuSO_4_.5H_2_O] 249.69 g/mol (Alpha chemi, India). Cefixime working standard [C_16_H_15_N_5_S_2_O_7_] 453.44 g/mol was kindly supplied by UP Pharma (Assuit, Egypt). The dry *Quinoa* seeds were purchased from the local market (Assuit, Egypt). Phosphoric acid is 85%, Sodium dihydrogen phosphate, Hydrochloric acid, and Sodium hydroxide (Alpha chemi, India). Buffer phosphate was prepared using about 16.8 g of sodium dihydrogen phosphate in 700 mL of deionized water with 0.5 mL of phosphoric acid at 85%.

### Methods

#### Aqueous extract preparation

The dried Quinoa seeds were mashed to a microparticle diameter of fewer than 1 μm. The aqueous extract was prepared in a concentration of 60% *wt/vol* in purified water then the solution was boiled using a reflux system at 1000 rpm for 15 min. Subsequently, the extract was filtered using the Whatman filter paper number 1 and reserved in the refrigerator to further use.

#### The green synthesis of *Cu NPs*

The green preparation of *Cu NPs* was prepared via the addition of the CuSO_4_.5H_2_O solution “3.5% *wt/vol* in purified water” to an aqueous *Quinoa* extract drop by drop at a ratio (1:6) at a temperature of 80 °C, 1200 rpm for 3 h. The biosynthesized NPs were left for 1 day. The mixture content was centrifuged at 10000 rpm three successive times for 15 min. Finally, the produced NPs washed 3 times using purified water through centrifugation at 10000 rpm three successive times for 15 min then the wetted NPs were heated at 80 °C for 1 h.

### Characterizations

The structure of annealed the as-prepared *Cu NPs* was conducted via a Philips X-ray diffractometer [PW 1710] at two theta scans in the range of 4–100 degrees with wavelength (λ) 1.541838 Å “Cu” (PW 1710, anode material Cu, at a voltage of 40 kV, current of 30 mA, optics: automatic divergence slit, beta filtering using graphite, monochromator).

The XRD parameters and crystallite size “D” could be determined using the Scherrer equation for each peak and the average crystallite size could be estimated as follows:

1 $$D \, = \, 0.9\lambda /\beta \, cos\left( \theta \right)$$

where ***D*** is the average crystallite size, ***λ*** is the wavelength for the used XRD radiation source which equals = 0.1541838 nm, ***β*** is the corrected full widths at half maxima of the measured peaks, and ***θ*** is the Bragg’s angle diffraction.

Also, the other XRD parameters could be estimated as the strain of the lattice (ℇ), placing distance (d), dislocation density (δ), and stacking fault (α) as follows [1, 6, 21, 22, 35]**:**

2 $$\varepsilon_{{}} = \beta /4 \, tan\left( \theta \right)$$

3 $$d \, = \, \lambda \, / \, 2 \, sin\left( \theta \right)$$

4 $$\delta \, = \, 1/D^{2 } in \, lines/nm^{2}$$

5 $$\alpha \, = \, \left[ {\left( {2\pi^{2} } \right)/\left( {45\left( {3 \, tan\left( \theta \right)} \right)^{0.5} } \right)} \right] \, \beta$$

6 $$Crystallinity \, \left( \% \right) \, = \, C_{A} /T_{A} x \, 100 \, \left( \% \right)$$

C_A_ is the summation of the crystalline peak areas in the diffractogram.

T_A_ is the summation of the crystalline and amorphous peak areas and noises in the diffractogram.

FT-IR analysis was recorded on a Thermo Fisher [Nicolet iS10 FT-IR spectrometer] in a wavenumber range of 4000–400 cm^−1^ using a potassium bromide disc technique. UV–vis absorption analyses were recorded in the range of 200–900 nm using PerkinElmer [LAMBDA 40 UV/Vis] Spectrophotometer using a quartz cell of 1 cm path length at room temperature.

The direct bandgap energy of the as-prepared *Cu NPs* could be determined using the Tauc equation as follows:

7 $$\left( {\alpha \, h\upsilon } \right)^{1/n} = \, C \, \left( {h\upsilon - \, E_{g} } \right)$$

where; *α* acts as the absorption coefficient, hυ represents the photon energy. *n* is the assumed value of n = ½ for direct allowed transition and n = 2 for indirect allowed transition, C is the independent energy factor, and E_g_ is the bandgap energy [47, 48].

The electrical conductivity at the temperature range 20–200 ºC was conducted using a high-resistor-meter [HP 4329A]; the electrical conductivity determinations for the *Cu NPs* were investigated as a sandwich electrode structure form where the silver coatings were used as electrodes (Ohmic contact).

A plot of EC at the logarithmic scale against the invert of the temperature in kelvin as the Arrhenius model suggested as manifested in the following equation:

8 $$log \, (\sigma ) \, = \sigma_{ \circ } ( - \, E_{a} /2.303K_{b} T)$$

where σ is the EC, σ。is the dielectric relaxation parameter caused by the localized electric charge carriers, E_a_ represents activation energy; K_b_ acts as the Boltzmann constant and T represents the applied absolute temperature.

The morphology of the *Cu NPs* and *Xim@Cu NPs* were investigated using scanning electron microscopy [SEM; JSM IT 200] and transmission electron microscopy [TEM; JEOL JEM-100C XII)].

The chemical elemental analysis of the as-prepared NPs was assessed using EDX analysis.

The crystallinity index could be estimated using the following equation [20].

9 $$CI \, = \, I_{1} /I_{2}$$

I_1_ acts as the particle size obtained from TEM analysis.

I_2_ acts as the crystallite size for the principal peak of “Miller indices at hkl" obtained from XRD analysis using the Scherrer equation.

*Xim* assay analysis was implemented using the LC-20A HPLC instrument with the PDA (Shimadzu).

Karl Fischer titration (KFT) [701–703 KF titrinio] was used for the semi-micro determination of water. The water percentage could be estimated through a reaction as oxidation/reduction of the Karl Fischer reagent. This reagent consists of a mixture of iodine, sulfur dioxide, and resin containing nitrogen atom that has a lone pair of an electron at least free from pyridine [1]. The as-biosynthesized *Cu NPs* mass of about 50 mg was introduced to the KFT in presence of the methanol as a reaction medium. The water percentage could be determined according to the following equations:

10 $$Titer \, \left( {mg/mL} \right) \, = \, \left( {mg \, of \, added \, water \, standard} \right)/\left( {mL \, of \, KFT} \right)$$

11 $$Water \, \left( \% \right) \, = \, \left( {Titer \, x \, mL \, of \, the \, KFT \, for \, Cu \, NPs \, x \, 100} \right)/\left( {Cu \, NPs \, mass} \right)$$

### Contaminated *Xim*-water treatment/removal activity using* Cu NPs* (nano bioadsorbent)

As a standard operating procedure of batch adsorption mode experiments, the parafilm was used to maintain the *Xim* drug adsorbate from evaporation during the adsorption process. To confirm the consistencies and the accuracy of the resultant data according to the guidelines of repeatability and validation, triplicate measurements of the test experiments procedure were carried out [49–53]. For *Xim* determination before and after the adsorption equilibrium process, the previous assay method by Al-Hakkani et al. was assessed [25]. The adsorbate dissolved completely in methanol. After each adsorption test, filtration using a nylon filter of 0.45 µm was conducted. As a confirmation procedure, further filtration was introduced using a syringe filter of 0.2 µm before the HPLC test injection.

#### Determination of the pH of the reaction solution

To determine the most fittable solution pH of the reaction according to previous studies, the (1–5) solutions pH range was conducted for getting the highest removal of *Xim* [33]. For the removal amount of *Xim *via* Cu NPs* the following equation was implemented:

12 $$Q_{{Xim}} = \left( {A_{i} {\text{ }} - A_{f} } \right)xV/M$$

***Q***_***Xim***_ is *Xim* adsorbed amount (mg/g); ***A***_***i***_ and ***A***_***f***_ are the initial and final assay of *Xim* (mg/L); V is the volume of the tested *Xim* solution (mL); ***M*** is the mass of *Cu NPs* (mg).

13 $$Removal\left( \% \right) \, = \, \left( {A_{i} - \, A_{f} } \right) \, / \, A_{i } x \, 100$$

All of the reaction parameters were kept constant at a temperature of 25 ºC, 25 mL of the *Xim* adsorbate concentration of 100 mg/L, and 30 mg of *Cu NPs* at fixed stirring at 300 rpm over 360 min. Solution pH adjustment was realized using NaOH or HCl at 0.1 M to reach the desired pH.

#### Effect of the *Cu NPs* adsorbent mass dose

Different masses of the *Cu NPs* (0.2–4.0) g/L were implemented and all of the adsorption process parameters were kept at a temperature of 25 ºC, 25 mL of the *Xim* adsorbate concentration 100 mg/L at pH 4.0 with fixed stirring at 300 rpm over 360 min.

#### Isothermal study and impact of the adsorbate *Xim* concentration

To get the convenient isothermal type of adsorption process; the following procedures have been done. Different 25 mL of the *Xim* adsorbate concentrations (50–200) mg/L at pH 4.0 at temperature 25 ºC, 30 mg of *Cu NPs* with fixed stirring at 300 rpm over 360 min. The result data were investigated according to two isothermal models as the following equations:

##### Langmuir model:

14 $$A_{f} / \, Q_{Xim} = \, \left( {1/q_{L} K_{L} } \right) \, + \, \left( {1/q_{L} } \right) \, A_{f}$$

15 $$R_{L} = \, 1/ \, \left( {1 + K_{L} C_{max} } \right)$$

**q**_**L**_ is the monolayer adsorption capacity of *Cu NPs* (mg/g); **K**_**L**_ represents Langmuir energy of adsorption constant (L/mg); **R**_**L**_ is the separation factor; **C**_**max**_ acts as the highest initial *Xim* concentration in the solution (mg/L) [54].

##### Freundlich model:

16 $$log \, Q_{Xim} = \, log \, K_{F} + \, \left( {1/n} \right) \, log \, A_{f}$$

**K**_**F**_ represents the Freundlich adsorption capacity of *Cu NPs* (mg/g); **n** is the Freundlich constant characteristics of the system, indicating the adsorption intensity [55]

#### Thermodynamic and kinetic studies

Effects of the reaction temperature and *Cu NPs* contact time were investigated. Studies were implemented at different temperatures in the range of 25–40 ºC.

The result data were investigated according to the following equations to estimate the thermodynamic parameters:

17 $$\Delta G \, = \, - \, RT \, ln \, K_{c}$$

18 $$ln \, K_{c} =_{{}} - \, \Delta G/ \, RT \, = \, - \, \left( {\Delta H/ \, RT} \right) \, + \, \left( {\Delta S/R} \right)$$

19 $$K_{c} = \, C_{ads} /Q_{Xim}$$

**∆G** determines the free energy change (J/mol); **R** is the gas constant (8.314 J/mol K); **T** represents the absolute temperature (K); **K**_**c**_ describes the thermodynamic equilibrium constant; **∆H** determines the enthalpy change (J/mol); **∆S** represents the entropy change (J/mol K); **C**_**ads**_ acts the concentration (mg/L) of the adsorbed *Xim*.

The time intervals have been studied at (2–360) min maintaining other of the reaction conditions constant as previously reported 25 mL of 100 mg/L concentration of the *Xim* adsorbate at pH 4.0 at 30 mg of *Cu NPs* with fixed stirring at 300 rpm. The result data were investigated according to two kinetic models as the following equations:

##### Pseudo-first-order model is by the Lagergren model:

20 $$log(Q_{Xim} - \, q_{t} ) \, = \, logQ_{Xim} - \left( {K_{1} /2.303} \right) \, t$$

##### Pseudo-second-order by McKay and Ho model:

21 $$\left( {t/q_{t} } \right) \, = \, 1/ \, \left( {K_{2} Q_{Xim}^{2} } \right) \, + \, \left( {1/ \, Q_{Xim} } \right) \, t$$

**q**_**t**_ represents the amount of *Xim* adsorbed by *Cu NPs* (mg/g) at predetermined time interval t; **K**_**1**_ describes the rate constant of the pseudo-first-order adsorption process (min.^−1^); **t** is the time interval (min); **K**_**2**_ describes the rate constant of pseudo-second-order adsorption process (g/mg min) [56, 57]

#### Actual *Xim*-water removal treatment from pharmaceutical wastewater after direct production of *Xim*

The contaminated Xim-water samples (rinse) after the production process of the finished product containing *Xim* were collected from the pharmaceutical industry. Firstly, some physicochemical parameters were determined as conductivity, pH, total dissolved solids (TDS), and HPLC assay. In a 250 mL beaker; 100 mL of real contaminated pharmaceutical wastewater samples that were previously filtered using a nylon membrane filter of 0.45 µm, 1 mg/mL of the *Cu NPs* was added with stirring for 1 h at 350 rpm at the temperature of 40 ºC. Then, the sample was filtered via nylon filter paper of 0.45 µm, followed by an additional filtration step using a syringe filter of 0.2 µm before the determination of the *Xim* assay by HPLC.

### Antibacterial activity

The *Quinoa* extract, *Cu NPs*, and *Xim@Cu NP's* antibacterial activities were studied against two couples of bacterial strains. *Bacillus subtilis (B*. *subtilis)* & *Staphylococcus aureus (S. aureus)* as Gram-positive bacterial type. *Escherichia coli (E. coli)* & *Pseudomonas aeruginosa (P. aeruginosa)* as Gram-negative bacterial type. The bacterial strains were obtained from the Microbiology lab, UP pharmaceutical industrial, Assuit, Egypt. The activity was conducted as previously approached procedures [22] via Cefixime as a positive control in concentration (100 µg/mL) and Dimethyl sulfoxide (DMSO) as a negative control for suspension of the tested materials. Each *Quinoa* extract, *Cu NPs*, and *Xim@Cu NPs* were added to the wells at a concentration of (100 µg/mL). The incubation of the plates was conducted at 30° C for 24 h. The inhibition zones were examined in mm and recorded.

## Results and dissection

### XRD analysis

**Fig. 2:**
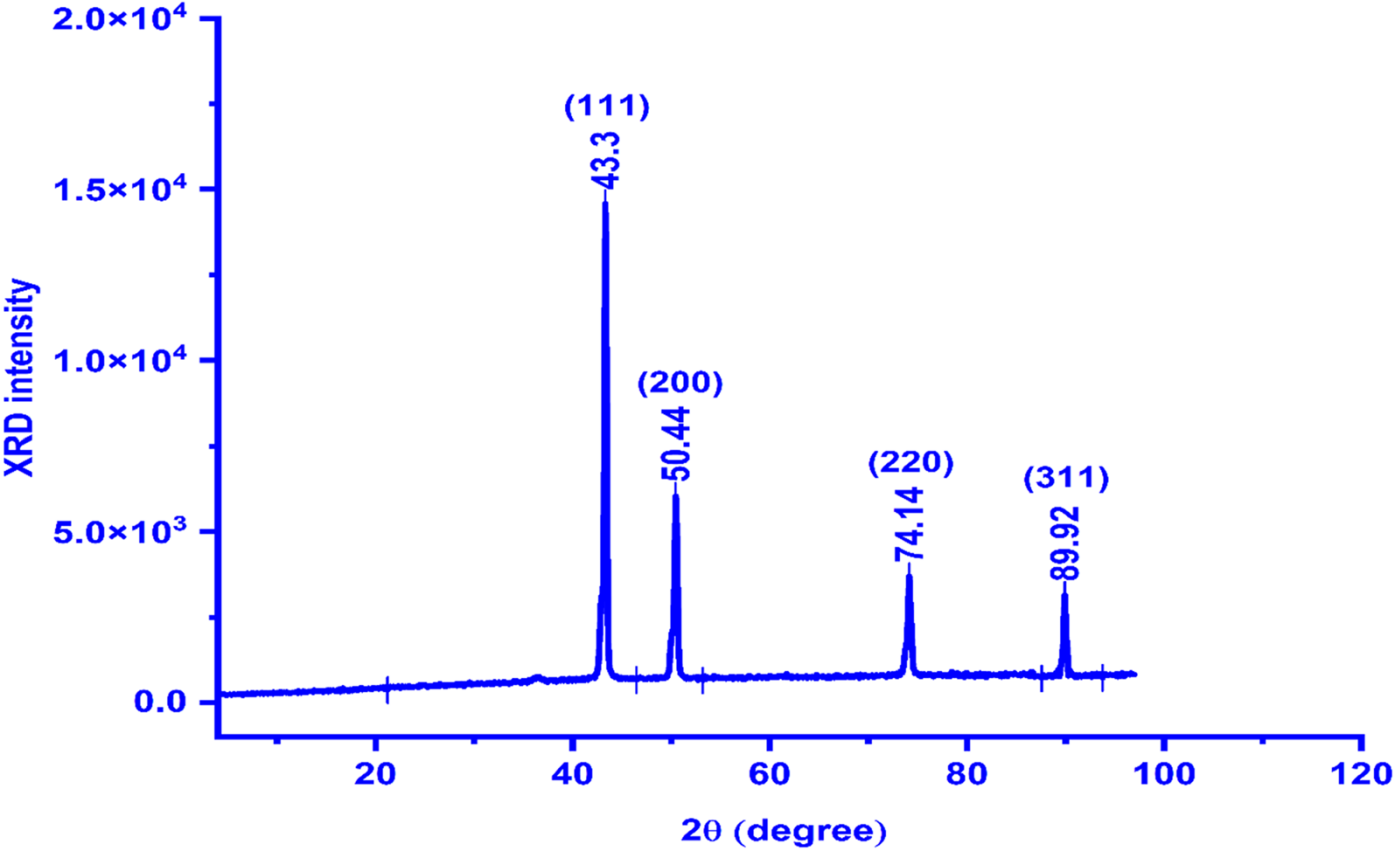
The as-biofabricated *Cu NPs* X-ray diffractogram

X-ray powder diffraction is the rapid and best tool technique to identify the material nature if it is crystalline or amorphous at the nanoscale [22]. Also, XRD is very useful to investigate the crystallographic system using comparison with the reference material cards. The XRD pattern of the *Cu NPs* ( Fig. 2) shows four principal peaks dedicated to the face center cubic system of the *Cu NPs* material according to reference card ICDD # 00–901-2043. Also, it was found to be agreed with recently previously reported works such as Al-Hakkani et al. [1] and Saddik et al. [33]. Miller’s indices (*hkl*) were be evaluated according to their two theta degree values and they found to be (111), (200), (220), and (311) which correspond to the two theta values 43.4º, 50.44º, 74.14º, and 89.92º respectively.

The most abundance referred to the crystalline peaks about 71.7% and the other percentage of 28.3% attributed to the noises in the XRD diffractogram indicating the high purity of the as-prepared *Cu NPs* [35, 58]. This result may be attributed to the capping and adsorption strength of bioactive constituents that are present in the *Quinoa* seeds aqueous extract [6]. These bioactive molecules are the main cause that prevents the as-prepared *Cu NPs* from further oxidation to any other copper oxide type [26]. The sharpness of the peaks is an indication of the crystallinity nature of the as-biosynthesized *Cu* NPs [1]. The average crystallite size “D” of the as-prepared *Cu NPs* was found to be 8.41 nm as shown in Table 1.

**Table 1:** XRD parameters of the as-prepared *Cu NPs* via *Quinoa* seed extract

Parameter	Peaks data			
Miller’s indices (*h,k,l*)	(111)	(200)	(220)	(311)
Measured 2θ (degree)	43.3	50.44	74.14	89.92
Reference 2θ (degree)	43.3162	50.4479	74.1236	89.9307
D (nm)	7.5786	7.7862	8.8282	9.4478
Average D (nm)	8.41			
δ (lines/m^2^)	1.74 × 10^16^	1.65 × 10^16^	1.28 × 10^16^	1.12 × 10^16^
Average δ (lines/m^2^)	1.41 × 10^16^			
ε	0.0124	0.0105	0.0065	0.0052
Average ε	0.009			
α	7.9 × 10^–3^	7.3 × 10^–3^	5.7 × 10^–3^	5.3 × 10^–3^
Measured d (nm)	0.2090	0.1809	0.1279	0.1091
Reference d (nm)	0.2087	0.1808	0.1278	0.1090

The smaller the particle size the higher the crystallinity [35]. So, according to the observed sharpness of the peaks, it could be said that we achieved and assured this assumption in our approach. Also, this result could be proved via the calculation of the percentage of crystallinity.

The crystallinity percentage was found to be 71.7% and this ratio is a rigid indication of our findings.

### FT-IR analysis

FT-IR analysis is the best tool to identify and confirm the presence of the possible functional groups of the bioactive constituents that are responsible for the reduction process of the copper metal ions [21]. FT-IR analysis has another important role in the confirmation of some of the functional group’s participation in the capping-stabilization role of the as-biosynthesized *Cu NPs*. Al-Hakkani et al*.* [3] and Jiang et al*.* [59] reported in their investigations that the content of the dried mass of most powder extracts is bioactive constituents such as flavones, polyphenols, flavonoids, and sugars. These bioactive molecules may be considered the main parameter that is responsible for the metal ions reduction in biosynthesized hematite and gold nanoparticles. After the bioreduction process, some of the bands in the extract were absent or deviated compared to before the reaction [21]. This may be attributed to the bioactive molecules such as polyphenols or flavonoids that are present in the plant extract that is involved in the bioreduction process.

These constituents are adsorbed at the surface of the NPs and gift the ability to further interaction between these NPs and some of the substances [4, 26]. The further interaction of these NPs may be attributed to the double bonds, hydrogen bonds, and or electrostatic interaction [22, 58].

**Fig.3:**
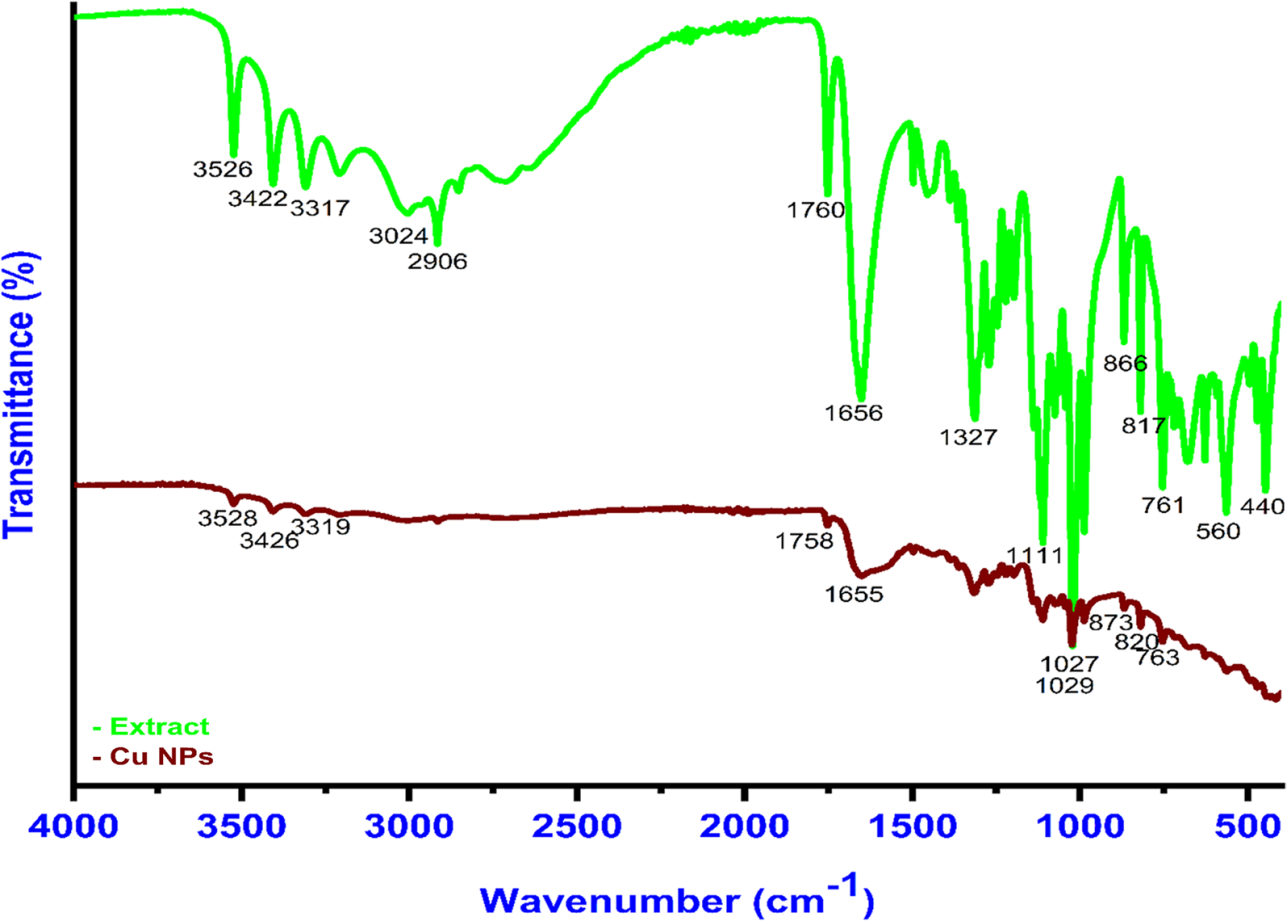
FT-IR spectra of the *Quinoa* extract and the as-biofabricated *Cu NPs*

As shown in (Fig. 3), the presence of many functional groups such as -CH at 3024, 2906 cm^−1^, -NH_2_ at 3526, 3422 cm^−1^, C = O at 1658 cm^−1^, and -OH at 1027 cm^−1^ in the *Quinoa* seeds powder plays an important role in the bioreduction, stabilization, and capping process [42–46, 60]. The absence of any characteristic Cu–O bands at vibrational modes at 614, 588, 534, and or 480 cm^−1^ that belong to cuprous or cupric oxides confirms the purity of the biosynthesized *Cu NPs* [61, 62].

#### The suggested mechanism of the as-biosynthesized Cu NPs

According to the obtained data from the previously reported studies, the suggested mechanism of the biofabricated *Cu NPs* could have occurred based on the bioreduction/complexation capability of the bioactive molecules that exist in the *Quinoa* seed's aqueous extract [63, 64]. Figure 1 depicted some of the identified *Quinoa* bioactive constituents. The formation of *Cu NPs* using *Quinoa* extract could be derived in the following steps:

Firstly, copper complex formation via any bioactive molecules such as aldehydes, carboxylic acid, phenols, amino acids, amines, or flavonoids compounds. These molecules could be gifted many free electrons that are present at the main core of our approach in the bioreduction process of the copper metal ions. This could be presumed by breaking the hydroxyl bond (-OH) in the bioactive molecule constructing a partial connection with the copper ion [29, 30].

**Fig.4:**
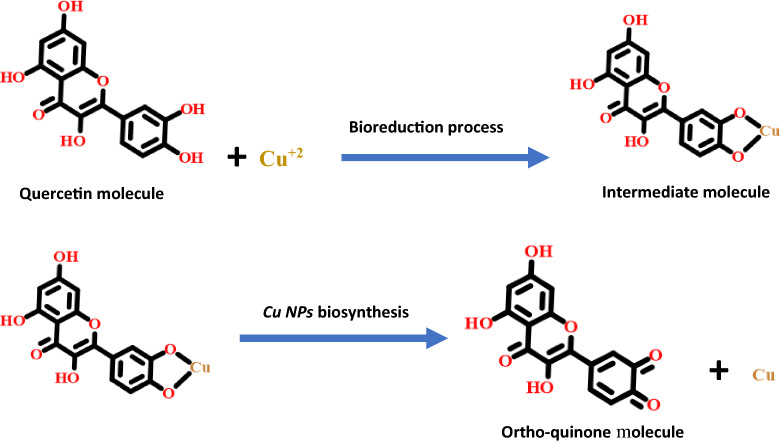
*Cu NPs* suggested bioreduction and biosynthesis mechanism via Quercetin in *Quinoa* seeds extract

Subsequently, a rapid breakage of these intermediated molecules and transformation of the copper metal ions in the bioreduction process into cuprous oxide NPs as an initiator precursor which is a rapid reduction step converted to *Cu NPs* at a zero-valent state depending on the strength of the bioactive molecules that could reduce the Cu^+2^ precursor ions. Finally, the bioactive molecule could be oxidized into the ortho-quinone position Fig. 4).

The biosynthesized *Cu NPs* are very active, unstable, and tend to oxidation, but the second role of the plant extract as a capping and stabilization agent appeared here forming an adsorbed layer of the bioactive constituents that protected the *Cu NPs* from further oxidation to any oxidation state as CuO or Cu_2_O NPs. So, the minor source content of the oxygen, nitrogen, sulfur, and/or carbon that may be present in the biosynthesized *Cu NPs* confirmed the stabilization and encapsulation process and this was confirmed by FT-IR analysis [1, 4, 6]. Also, this assumption could be confirmed using EDX analysis as it was manifested in our investigations [35].

### UV–Vis. analysis

**Fig. 5:**
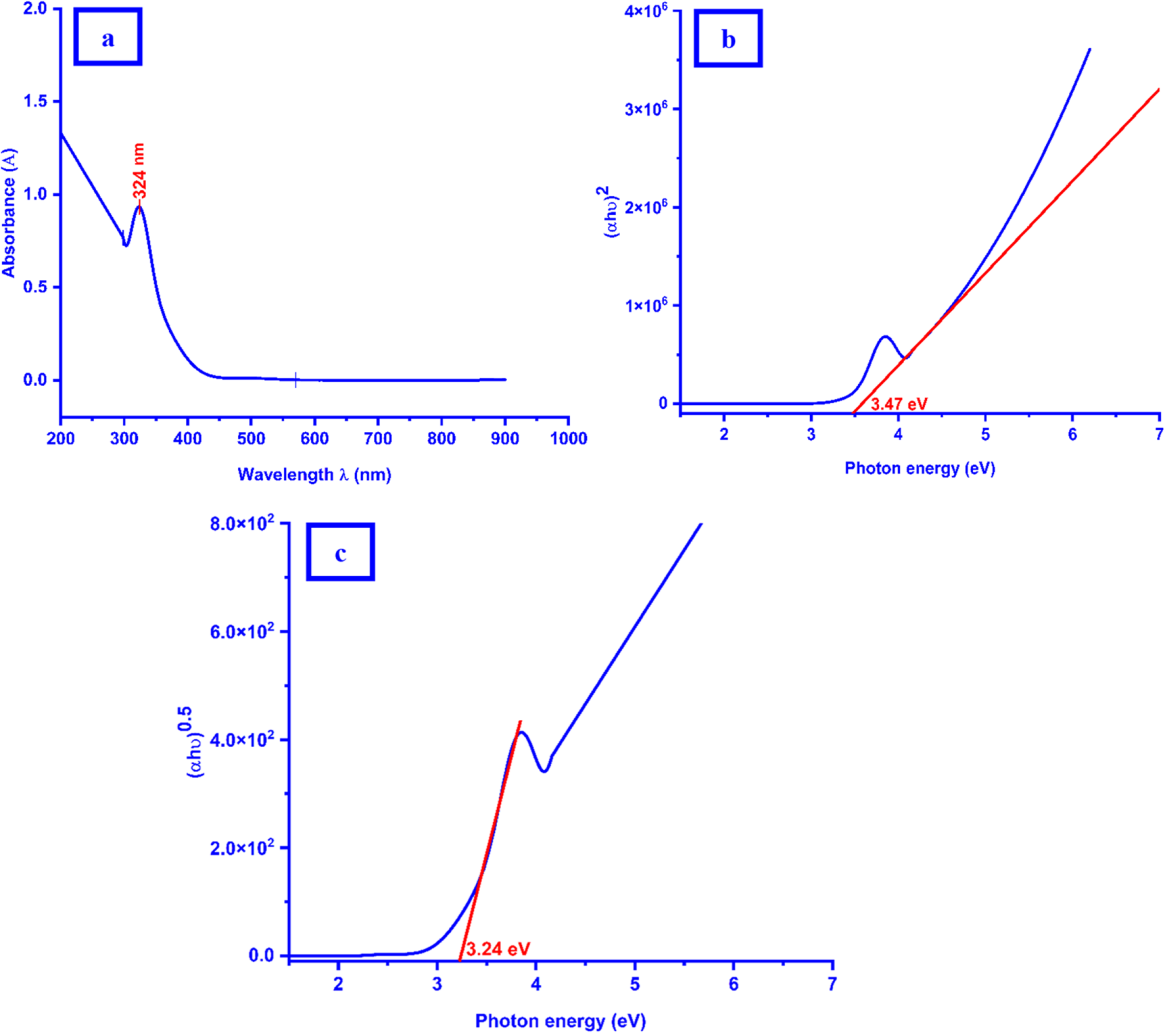
Spectra of the as-biofabricated *Cu NPs* **a** UV–Vis. excitation absorption, Tauc plots of energy bandgap **b** Direct transition, **c** Indirect transition

According to the primary monitoring, there are color change of the copper (II) ions was conducted from pale blue to a brown color precipitate accompaniment with a characteristic smell directly after the addition of the copper (II) salts solution to the *Quinoa* seeds liquid extract indicating the formation of *Cu NPs*. The green synthesized *Cu NPs* powder sample was suspended in the purified water and the optical characterizations were investigated. There is no presence of the copper (II) broad peak that appeared about at 812 nm in the visible region after the addition of the *Quinoa* seeds liquid extract confirming that the reduction reaction was done. The *Cu NPs* exhibited one absorption peak in the UV range at 324 nm (Fig. 5a).

This result was found to be agreed with the previous investigation of the surface plasmon (SPR) peak at 320 nm conducted by Kalpana et al*.* [65]. The stability of the as-biosynthesized *Cu NPs* was checked over 7 days as a suspension form in the purified water with monitoring the presence of the dedicated SPR peak in the spectrum.

In general, the classification of any material could be described as a semiconductor if it's E_g_ less than 5.0 [1].

The E_g_ in the direct transition was determined by extrapolating the linear portion of the plot of (αhυ)^2^ against hυ (Fig. 5b) and it was found to be in the range (3.47 eV) where the indirect band gap energy was found to be 3.24 eV (Fig. 5c). It is clear that from the estimated value of the E_g_ for the direct transition, confirmation of the proposal of the crystallinity nature of the as-biosynthesized *Cu NPs*. This assumption was investigated by Reddy et al*.* [66] and Al-Hakkani [26, 35], who reported that the bulk E_g_ for copper is approximately 2.0 eV. As a result of the quantum confinement effect and *Cu NPs* size reduction; the increase of E_g_ maybe came back to the presence of intragap states [22]*.* So, as a result of the determined E_g_ value, the as-prepared *Cu NPs* may be used as a semiconductor material according to their good optical properties. Also, Al-Hakkani [1, 22] and Zimmermann [67] and their co-authors reported that if the direct band gap energy is higher than the indirect band gap energy, the allowed transition is the direct bath.

### Electrical conductivity (EC)

**Fig. 6:**
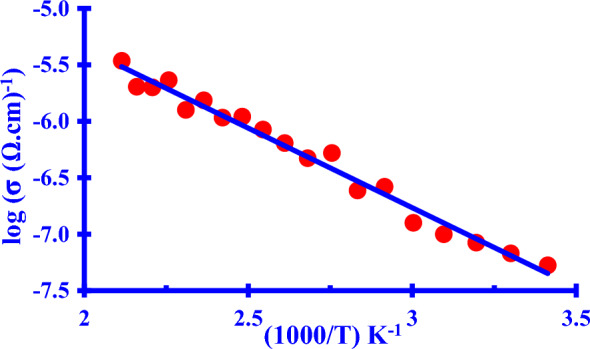
Arrhenius relationship of the electrical conductivity of the as-prepared *Cu NPs*

The influence of Cu NPs EC on the temperature change is a linear relationship (Fig. 6) The increase in temperature was accompanied by an increase in the EC in the applicable range.

So, the as-prepared *Cu NPs* have a semiconductor material behavior [1, 3, 22, 26]. According to the output data using the Arrhenius equation at room temperature; the conjugated resistivity (ρ) was found to be 1.88 × 10^7^ *Ω*.cm at 293 K temperature with an σ = 5.31 × 10^–8^ *μ*S/cm. The activation energy (E_a_) was estimated and it was found to be 0.28 eV.

This assures of the quantum condiment effect of the as-biosynthesized *Cu NPs* at the nanoscale [33] and our previous findings of optical investigations of the UV–Vis investigation. The smaller value of (E_a_) proves that the as-prepared *Cu NPs* could be used in photo-electronic applications as micro transistors where it is very easy to excite.

### TEM analysis

**Fig. 7:**
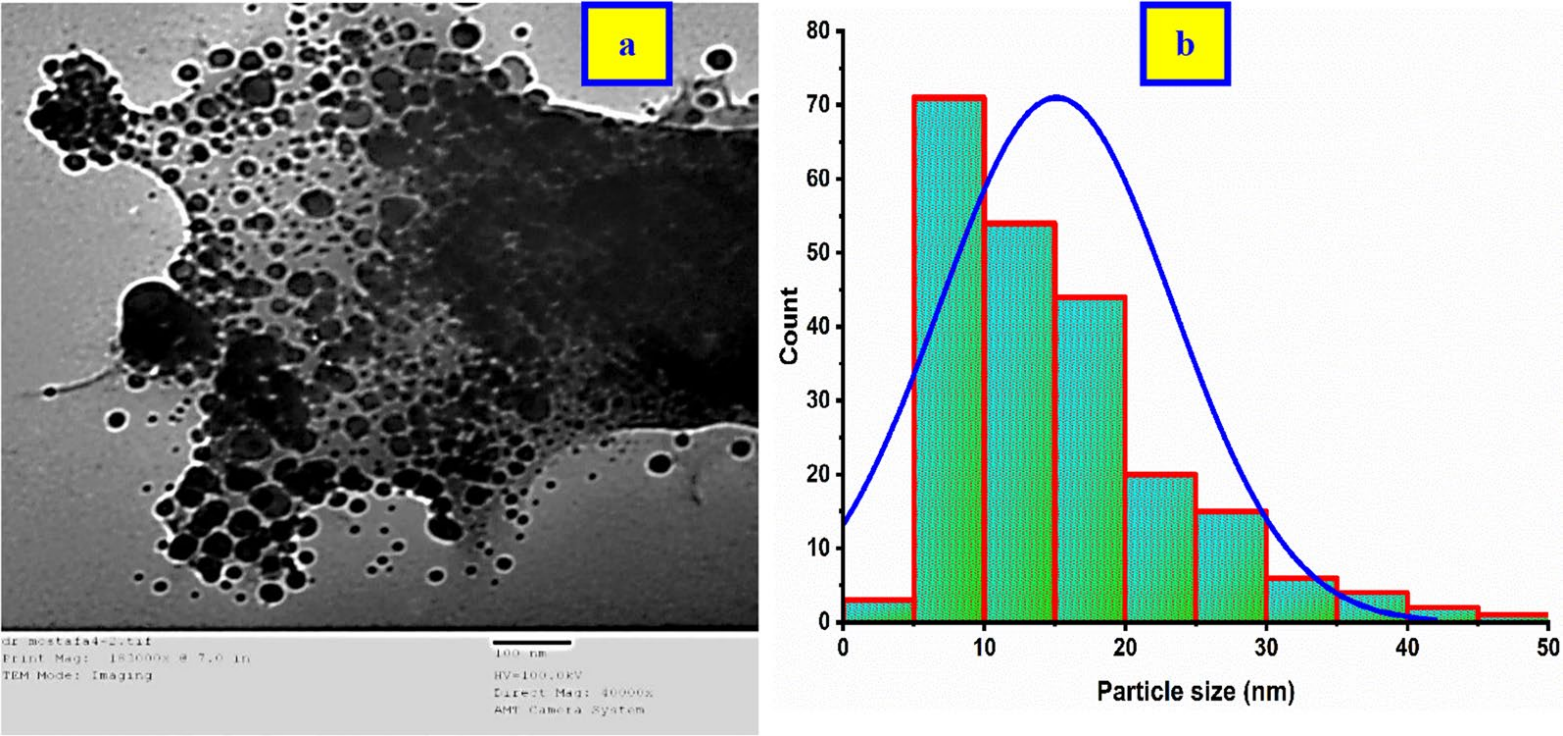
The as-biofabricated *Cu NPs* **a** TEM image, **b** Particle size distribution

The TEM image of the as-prepared *Cu NPs* **(**Fig. 7a**)** depicted spherical and cubic shapes that appeared as polycrystalline particles without intensive agglomeration. The average particle size of *Cu NPs* was estimated using the Image J software program at 220 locations and it was found to be 15.1 ± 8.3 nm showing a minimum particle size of 3.8 nm and a maximum particle size of 49.2 nm with a median was 13.3 nm as the corresponding histogram figure of the particle size distribution as shown in (Fig. 7b).

This finding well was found compatible with the XRD results that confirm the formation of the NPs at the nanoscale.

### The crystallinity index* (CI)*

The crystallinity index investigation has a necessary effect on morphology identification [22]; where it assured a high crystallinity of the conducted material. if the CI value is slightly greater than but close to the unity with monodispersibility nature. On the other hand, if the CI value has much more than the value of 2,3, …; it indicates the polycrystallinity material. The calculation of CI was conducted according to Eq. (9) The particle size obtained from TEM analysis was (15.1 nm), and the crystallite size for the principal peak of "Miller indices at hkl (111)" was obtained from XRD analysis using the Scherrer equation was (7.6 nm). So, the calculated CI ≈ 2.0 where confirms the polycrystallinity of the as-prepared *Cu NPs *[20]. The high crystallinity has a very vital contribution to achieving the enhancement in the electrical properties of the semiconductor [35].

According to the obtained results from XRD, and TEM analyses of the as-biosynthesized *Cu NPs*; it was confirmed that the high degree of crystallinity of the material under examination. This property could contribute to the enhancement of the excitation of the electrons” photo-excitation induction” and suppress charge carriers from the recombination that may be improved the catalytic activity of *Cu NPs *[26].

### SEM analysis

In general, most of the properties of NPs are depended on their shape and size. The higher the specific surface area, the smaller the particles [20, 22]. These properties consider the fulcrum to study for most nanoparticle applications as anticancer and antibacterial potentials [1].

**Fig. 8:**
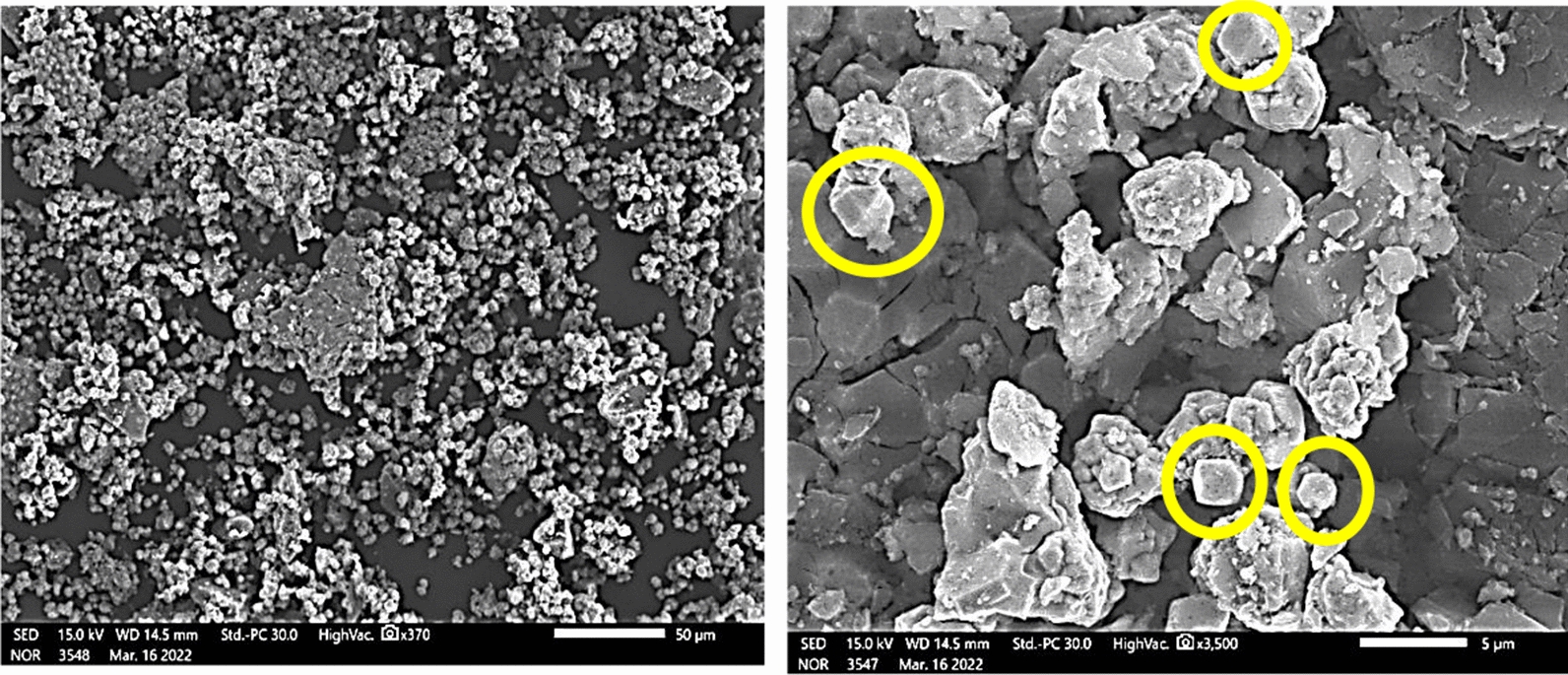
The as-biofabricated *Cu NPs* SEM images

SEM morphologies of the as-prepared *Cu NPs* confirm the results of XRD and TEM analyses where they appeared as agglomerated cubic and spherical shapes (Fig. 8).

This agglomeration may be attributed to the bioactive constituents that are adsorbed onto the as-prepared *Cu NPs* [1, 22]. So, it is a strong confirmation of the capping activity of the *Quinoa* seeds constituents that were surrounded by the formed *Cu NPs*. The occurrence of the agglomerations may be attributed to the formed steric hindrance effect between the bioactive contents in *Quinoa* seeds extract and the formed *Cu NPs*.

In the SEM figure (Fig. 8) we hinted at two captures one of them at 50 μm as a surface screen then we took a focused image at a 5 μm scale to demonstrate the cubic shape.

Table 2 summarizes the literature survey comparisons among different plant extracts used in green biofabrication of *Cu NPs,* particle size, and morphological characterization. Where it is revealed that the main cause of the diversity of the formed nanoparticles in particle sizes, and morphology is attributed to using of the different plant parts and/or plant extracts [1, 26].

**Table 2:** Particle sizes and shapes comparison among different plant extracts used in green biofabrication of *Cu NPs*

*Plant*	Particle size (nm)	Shape	References
*Tilia*	4.7–17	Spherical & cubic	[27]
*Euphorbia nivulia* stem latex	5–10	Spherical	[68, 69]
*Datura innoxia* aqueous leaves	5–15	Spherical	[70]
*Syzygium aromaticum “Clove”*	5- 40	Spherical & granular	[71]
*Plantago asiatica* leaf	7–35	Spherical	[72]
*Syzygium aromaticum* bud	12	Spherical	[73]
*Triumfettarotundifolia*	12.46	Like triangle	[74]
*Quinoa seeds*	15.1 ± 8.3	Cubic	Current study
*Calotropis procera* latex	15 ± 1.7	Spherical	[75]
*Ginkgo biloba L.* leaf	15–20	Spherical	[76]
*Punica granatum* peel	15–20	Spherical	[77]
*Guava*	15–30	Flakes	[78]
*Phyllanthus embilica “Gooseberry”*	15–30	Flake	[79]
*Citrus grandis* peel	22–27	Spherical	[80]
*Pineapple*	30–50	Cubic	[81]
*Cassia Auriculata* leaf	38.1–43.5	Spherical	[82]
*Aloe vera* flower	40	Spherical	[83]
*Arevalanata* leaves	40–100	Spherical	[84]
*Lemon* fruit	45	Cubic & rod	[85]
*Magnolia Kobus* leaf	45–110	Spherical	[86]
*Azadirachta indica* leaf	48	Cubic	[87]
*Licorice*	50.25 ± 9.20	Cubic	[33]

### EDX analysis

**Fig. 9:**
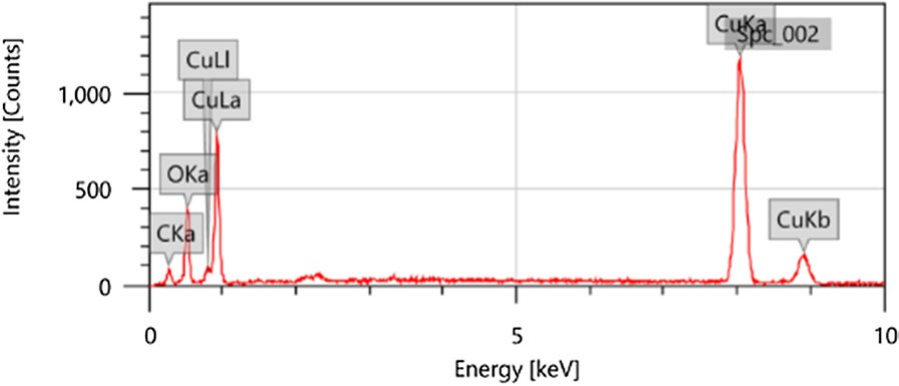
EDX analysis of as-prepared *Cu NPs*

The chemical analysis was investigated using EDX analysis at an acceleration voltage of 25.0 kV. The result confirmed the biosynthesized *Cu NPs* content contained only carbon, oxygen, and copper referring to the purity of the as-prepared NPs (Fig. 9). The source of carbon, and oxygen is the bioactive constituents present in the *Quinoa* seeds extract that is the main contributor in the biosynthesis reduction process that confirmed the capping and stabilization of the formed NPs [1, 20].

The strong intensity, sharpness, and narrowness of the peaks assured the crystallinity nature of the biosynthesized NPs [4]. The output data was found to be compatible with XRD and FT-IR analyses. As the presence of a minor content of carbon and oxygen is another evidence of the encapsulation and stabilization role of the bioactive constituents in the *Quinoa* seeds extract. Many recent studies reported this phenomenon confirming the adsorption of some organic bioactive constituents at the surface of the as-prepared metal nanoparticles as *Cu NPs* [64, 88–93]. The presence of the minor content of oxygen is not a confirmation of the CuO or Cu_2_O NPs where this indication was assured by XRD analysis that exhibited only the diffraction peaks dedicated to *Cu NPs*. Additionally, FTIR did not manifest any transmittance vibrational bands for CuO or Cu_2_O NPs in its spectrum as reported by several approaches [93].

### Water content determination

The water/moisture content using a semi-micro method could be used to determine the water content which may be presented as moisture or crystalline water expressed in percentage units. This method is very common in use, especially in water content determination for pharmaceutical drugs and food supplements either in solid or liquid forms [22]. The water content using the KFT method was determined and it was found to be 5.78%. This result confirms the polycrystallinity nature of the biosynthesized using TEM, SEM, and EDX analyses especially since we did not use calcination for the formed nanoparticles.

### Cefixime wastewater remediation via* Cu NPs* (batch adsorption examination)

#### Determination of the pH of the reaction solution

**Fig. 10:**
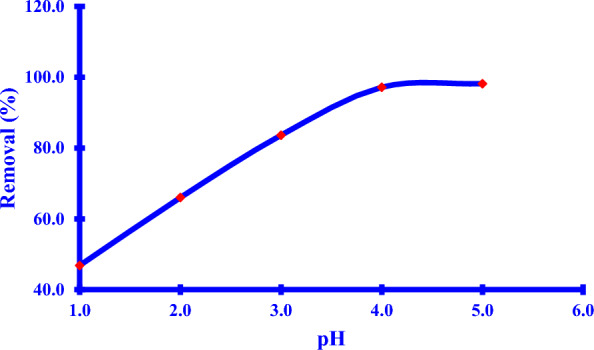
*Xim* removal (%) against pH solution change effect

The pH of the *Xim* adsorbate solution has a strong effect on the adsorption process which has a direct impact on its solubility and dissociation which simplifies the interaction of the relationship with the adsorbent [58]. At a highly acidic medium less than pH 2.1, the *Xim* exists as a protonated ion when an amine group gained a proton causing the lower removal of the adsorption where the *Xim* ions exist as cations [4]. An increase of the pH at 2.1 > pH > 2.92 causes the presence of the zwitterion ion that is lead to a relatively small increase in adsorption rate; where the carboxylic group in the *Xim* nucleus is deprotonated. After the rise of the pH solution higher than 3.45; the increase of the adsorption rate reached the maximum of about pH 4 (Fig. 10). That is because of the presence of the deprotonated two carboxylic groups and so, more negative anions were formed. This could be attributed to the electrostatic attraction force between the *Cu NPs* surface and *Xim* anions [21, 94].

Another assumption could be considered as a result of the molecular structure of the *Xim*. Nobody can deny the potential interaction using π-π interaction between the functional groups that can be occurred at the *Cu NPs* surface during the green synthesis and the *Xim*; also, hydrogen bonds could be produced as the *Xim* contains carbonyl, hydroxyl, and amino groups.

#### Effect of the adsorbent dose

**Fig. 11:**
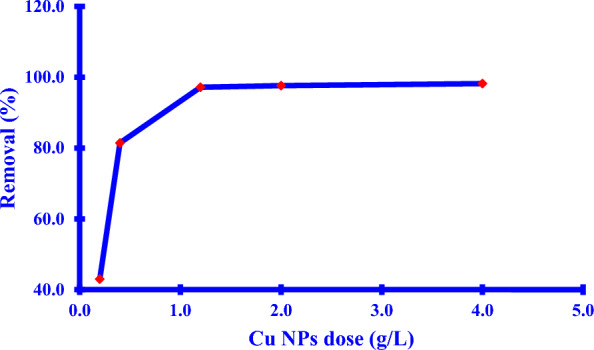
*Xim* removal (%) against adsorbent of *Cu NPs* mass effect

A significant impact of the *Xim* removal using *Cu NPs* as shown in (Fig. 11) manifested progress in the adsorption removal of the *Xim* by the increase of the *Cu NPs* dose up to 1.2 g/L. This increase could be attributed to the higher surface areas of the *Cu NPs* that generated a large number of the available active sites for adsorption [20].

When the Cu NPs mass was small, the binding capturing regions on the Cu NPs surfaces were constrained and not large enough to accomplish the high adsorption of the Xim, resulting in small adsorption performance. An increase in the total mass of the dosage of Cu NPs resulted in the creation of the active sites for Xim binding, confirming that more Xim was adsorbed on the surfaces of Cu NPs. Any increment in mass beyond 1.2 g/L of Cu NPs was associated with a slight improvement in Xim adsorption.

#### Effect of the *Xim* concentration (isothermal study)

**Fig. 12:**
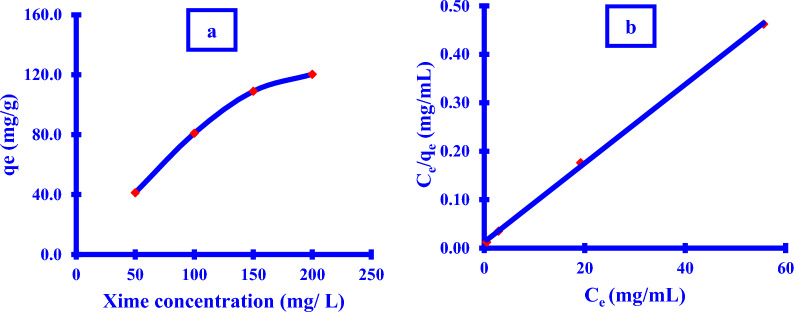
Effect of the **a**) *Xim* concentration; **b**) Langmuir isothermal model

(Fig. 12a) demonstrated an increase in the adsorption of Cu NPs by the Xim at various concentrations. When the concentration of Xim was increased from 50 mg/L to 100 mg/L, the adsorption capacity increased from 41.3 mg/g to 81.0 mg/g. Following that, a straightforward rise in the adsorption rate of 81.0 109.0 120.3 mg/g was shown.

The parameters of the different adsorption models were analyzed in Table 3 to evaluate the most convenient isothermal model that can describe the relationship between the *Cu NPs*/*Xim* in the present study.

**Table 3:** Isothermal model parameters

Item	Isothermal model	
	Langmuir	Freundlich
R^2^	0.9994	0.9232
Model parameter	q_L_ = 122.9	n = 4.5
	k_L_ = 0.654	1/n = 0.222
	R_L_ = 0.008	k_F_ = 54.2

Langmuir was considered to be the most appropriate isothermal model. This result was based on a comparison of the "R^2^" regression analysis for the two models, which was close to the unit (0.9994). (Fig. 12b). The reported Langmuir qL (122.9 mg/g) was found to be very equivalent to the measured qe (120.3 mg/g). This indicates that the Xim adsorption onto the Cu NPs substrate surface followed a monolayer building type on the Cu NPs surface via a homogeneous consistency in the energy for all active Cu NPs surface sites.

Furthermore, the adsorption experiment was found to be beneficial, with the Langmuir factor; 0 < R_L_ < 1 being 0.008 for the Xim concentration utilizing 100 mg Cu NPs at 298 K for 360 min.

Freundlich model may be used to confirm the adsorption process favorability through the n factor if it is ranged between 1 and 10 [21]; it is found to be equal to 4.5. As a result, the value of (1/n) was found to be 0.222; which revealed that the adsorption was not close to zero. This causes the adsorption not to follow the Freundlich model assumption as “heterogeneous for the surface energy of the binding active sites with reversible adsorption at multilayer formation”. The high k_F_ (54.2 mg/g) value indicated that a high adsorption capacity was carried out [21, 95].

The maximum capacities of *Cu NPs* and other adsorbents against *Xim* were presented in Table 4 to compare the advantages of using *Cu NPs* as a good nano adsorbent for the removal of *Xim* from contaminated wastewater. The as-prepared *Cu NPs* revealed a reasonable and acceptable maximum adsorption capacity. So, it could be used as a promising nano adsorbent for the *Xim* removal from contaminated aquatic environments in pharmaceutical industries and hospitals.

**Table 4:** The maximum capacities of *Xim* for different nano adsorbents

Adsorbent	Maximum capacity (mg/g)	Ref
Multi-Walled Carbon Nanotubes	820.0	[17]
Activated carbon by potassium hydroxide Activated carbon by sodium hydroxide	571.5 557.9	[18]
MgO NPs	526.31	[15]
Nano-sized activated carbon (pomegranate peel)	181.81	[94]
*α-HNPs*	147.1	[4]
*Cu NPs*	122.9	Current study
Cu-chitosan/Al_2_O_3_ NMs	30.5	[16]
Mg(OH)_2_	12.89	[19]

### Kinetic study

**Fig. 13:**
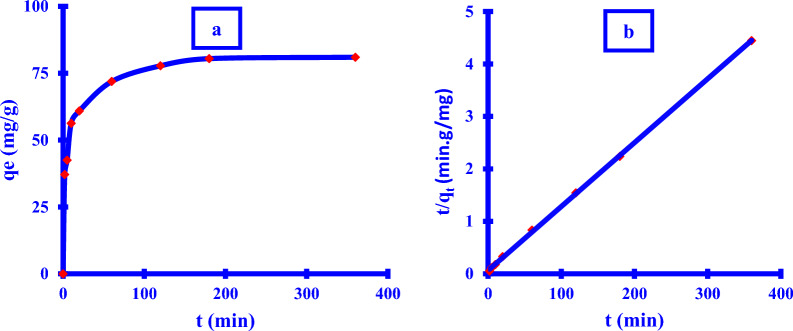
**a** *Xim* adsorption removal (%) against different time intervals; **b** McKay- Ho pseudo-second-order

The kinetic pathway of Xim adsorption onto the Cu NPs surface was reported to be as follows (Fig. 13a). The results revealed that Xim adsorption was directly proportional to the time. The majority of the Xim adsorption happened during the first sixty minutes of the procedure. After the initial 3 hrs, there was a small rise in Xim adsorption, and the adsorption process was unprofitable.

Because of the abundance of accessible free active binding locations on the Cu NPs surface, the adsorption process accelerated fast in the first 10 min. As a result, modulated unbonded active sites may be accessible at the surface of Cu NPs. A gradually enhanced adsorption rate in the Xim removal was seen between (60–180) min.

Table 5 manifested the obtained results of the different two kinetic models. It is clear to man that; the pseudo-second-order model is the most acceptable (Fig. 13b). The R^2^ was found to be close to the unit (0.9998), these findings confirmed the chemisorption process [39]. The estimation of the adsorption maximum capacity “q_e_ “value was found to be 82.1 mg/g which was very close to the experimental value of 81.0 mg/g after passing 6 hrs from the start of the adsorption process.

**Table 5:** Different parameters of the kinetic models

Item	Kinetic models	
	Lagergren pseudo-first-order	McKay & Ho pseudo-second-order
R^2^	0.9849 K_1_ = 0.023 Calculated q_e_ = 38.3	0.9998 K_2_ = 0.002 Calculated q_e_ = 82.1
Model parameter		
	Experimental q_e_ = 81.0	

The pseudo-second-order model supposed chemical adsorption could be occurred [4, 39]. The efficiency of the adsorption was found to be proportional to the number of free-active sites occupied via the *Cu NPs*. This finding confirms the obtained results of the isothermal study that supported the chemisorption occurrence and also, was agreed with the previously reported study conducted by Al-Hakkani and his co-authors [4]. Also, it was reported that the *Xim* maybe acts as a hexadentate ligand to produce a highly active complex with several metal ions such as Fe (III) ion, especially at high temperatures implementation. So, [Fe(*Xim*)(H_2_O)(Cl)]0.7H_2_O complex in an octahedral geometry could be formed [96]. Another suggested mechanism involves a non-electrostatic—dispersive force π- π as well as hydrophobic interactions between the *Xim* component and Cu NPs [39].

### Thermodynamic study

**Fig. 14:**
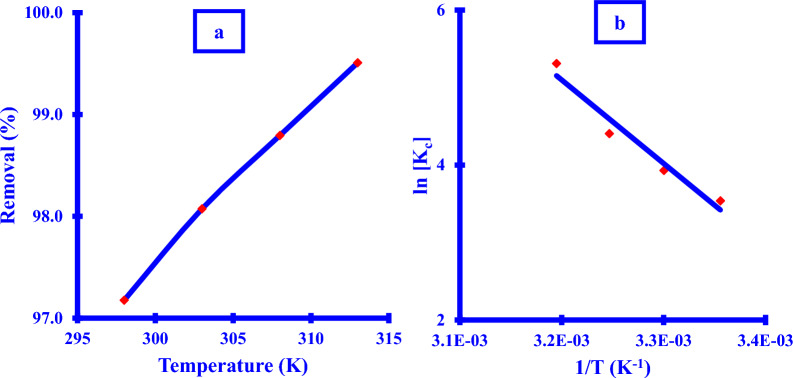
**a** *Xim* adsorption removal (%) against temperature, **b** Van’t Hoff relation of the *Cu NPs* adsorption process

The removal % was found to be directly dependent on the reaction temperature as shown in (Fig. 14a). *Xim* adsorption thermodynamic study via* Cu NPs* was implemented at different temperatures in the range 298–313 K to conclude the nature of the robustness and practicability of the adsorption process.

The maximum removal percentage of *Xim* was realized at the highest temperature that has been carried out in an endothermic process as shown in Table 6.

**Table 6:** Thermodynamic parameters of the *Xim* adsorption using *Cu NPs*

Temperature (K)	Removal (%)	ΔG (kJ/mol)	
298	97.2	− 8.8	
303	98.1	− 9.9	
308	98.8	− 11.3	∆H = + 89.5 kJ/mol
313	99.5	− 13.8	∆S = + 328.9 J/mol K

The association between temperature changes from 298 to 313 K and Xim adsorption removal was determined to be increased utilizing Cu NPs as the following trend, from 97.2% to 99.5%. This increase might be ascribed to the chemical interaction that developed between the active binding locations for the Cu NPs or the capping layer enclosing the Cu NPs during nano-phase biosynthesis, as described by Al-Hakkani et al. reported [4, 6].

Thermodynamic parameters were established based on the correlation between ln Kc and reaction temperature (Fig. 14b). The adsorption process is verified by the negative sign of the free energy change G. Furthermore, the presence of H and S demonstrated that the adsorption process was endothermic. As can be seen, raising the solution temperature causes an increase in the removal percent of Xim (Fig. 14a). The shift in the enthalpy function H was estimated to be 89.5 kJ/mol, which lies within the range of the chemisorption process of 80 kJ/mol [4] reflecting prior findings from isothermal and kinetic investigations.

### Actual *Xim*-water removal treatment from pharmaceutical wastewater after direct production of *Xim*

The characteristics and physicochemical parameters of wastewater and assay of the *Xim* before and after adsorption remediation were conducted and listed in Table 7. The working procedures revealed the high impact of the adsorption technique in the pharmaceutical wastewater remediation using as-biosynthesized *Cu NPs* as a promising nano adsorbent.

**Table 7:** Water physicochemical parameters and an assay of the *Xim* of the actual waste sample before and after adsorption treatment using the *Cu NPs*

Characteristic parameter	Before	After
Concentration (mg/L)	37.4	Not detected
Conductivity (μS/cm)	265.7	97.5
TDS (mg/L)	135.6	51.7
pH	9.32	7.03

### Antibacterial activity

**Fig. 15:**
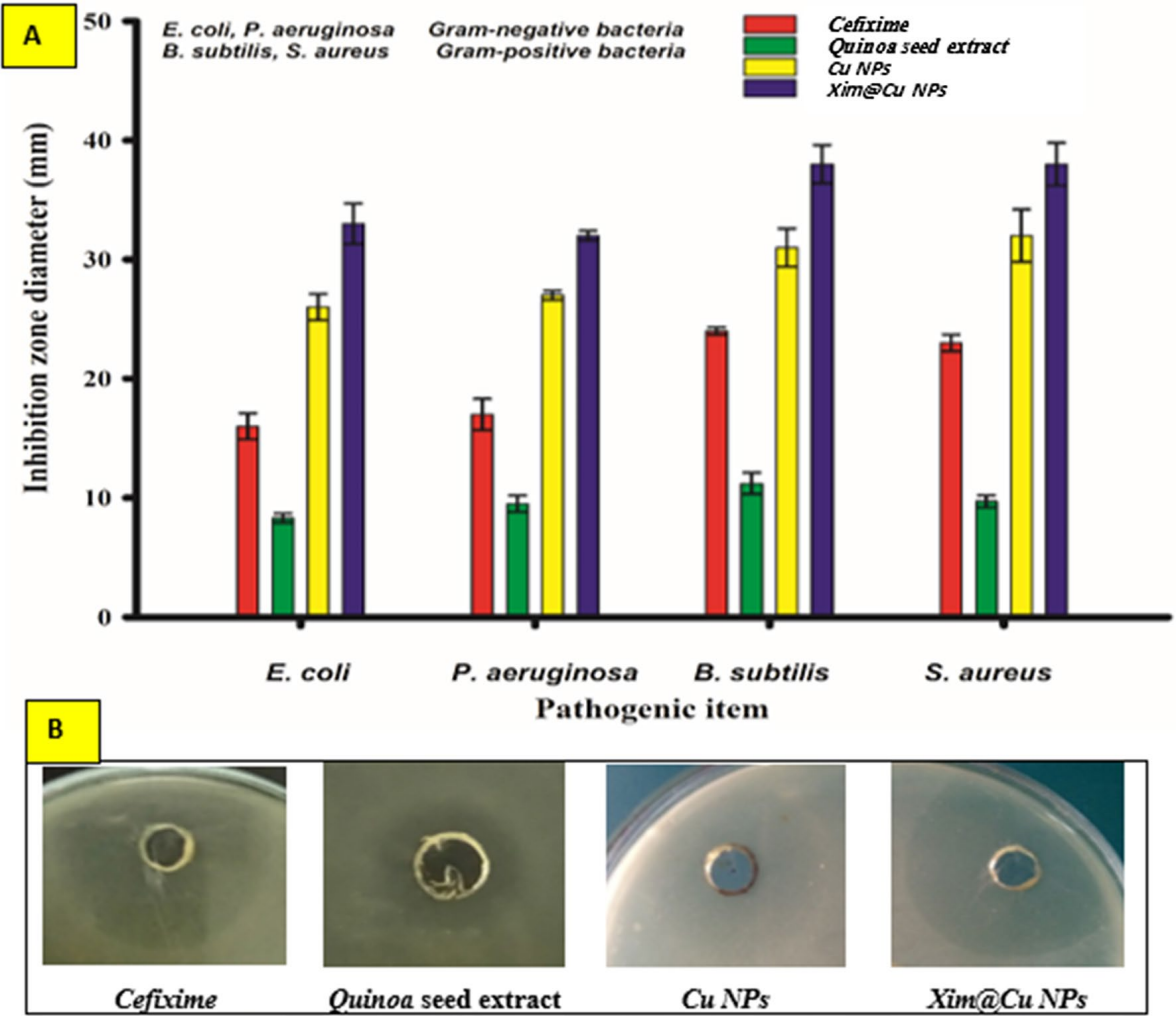
Comparison of the antibacterial activity of **A**) *Cu NPs* and *Xim@Cu NPs* among different microorganisms, **B**) Representative antimicrobial plate showing inhibition zone of the different treatments on *S. aureus*

The inhibition zones in mm were measured and recorded in Table 8 & (Fig. 15**)**. All of the tested materials were found to be effective as antibacterial with a directly proportional concentration of 100 µg/mL except DMSO which represented a negative control. The most potent microbial species against *Xim@Cu NPs* were found to be 33, 32, 38, and 38 mm for *E. coli, P. aeruginosa, B. subtilis,* and *S. aureus* respectively. The adsorbed *Xim* on the *Cu NPs* revealed a synergistic impact on either Gram-negative bacteria or Gram-positive bacteria compared with *Cu NPs* alone.

**Table 8:** Antibacterial activity of the *Cu NPs* & *Xim@Cu NPs* against different bacterial species

Item	Inhibition zone (mm)							
	*E. coli*		*P. aeruginosa*		*B. subtilis*		*S. aureus*	
Cefixime standard	16 ± 1.1		17 ± 1.3		24 ± 0.3		23 ± 0.7	
*Quinoa*	*8.3* ± 0.4		*9.5* ± 0.7		*11.2* ± 0.9		*9.7* ± 0.5	
NPs	*Cu NPs*	*Xim@Cu NPs*	*Cu NPs*	*Xim@Cu NPs*	*Cu NPs*	*Xim@Cu NPs*	*Cu NPs*	*Xim@Cu NPs*
	26 ± 1.1	33 ± 1.7	27 ± 0.4	32 ± 0.4	31 ± 1.6	38 ± 1.6	32 ± 2.2	38 ± 1.8

Moamen et al*.* demonstrated that the *Xim*/Fe(III) complex could be formed at high activity compared with *Xim* alone when investigated against bacterial species *E. coli, S. aureus*, *Proteus vulgaris*, *Klebsiella pneumoniae*, and *P. aeruginosa* [96]*.* The antibacterial activity of each of *Cu NPs* and *Xim@Cu NPs* may be attributed to the nanoscale nature of the formed nanoparticles that facilitate the penetration cell wall of the bacteria distorting the microorganism content [4, 21, 22, 35]. According to our observable results;we confirm the great strength of *Cu NPs* and *Xim@Cu NPs* as antibacterial agents, especially against *B. subtilis,* and *S. aureus* species as Gram-negative bacteria examples.

## Conclusions

The aim of this work is biofabrication of the copper nanoparticles via the Quinoa seeds extract green route for the first time. XRD results confirmed the synthesis of the pure crystalline face center cubic system of the *Cu NPs* with an average crystallite size of 8.41 nm. FT-IR spectroscopic analysis assured the bioreduction process, capping, and stabilization using *Quinoa* seed extract. UV–Vis. analysis was conducted to determine the absorption and surface plasmon resonance impact showing the absorption peak at 324 nm with an energy bandgap of 3.47 eV. Electrical conductivity was conducted assuring the semiconductor nature of the biosynthesized *Cu NPs*. TEM analysis also was used to assess the cubic shapes at a particle size of 15.1 ± 8.3 nm and crystallinity index about equal to 2.0. EDX analysis was conducted to confirm the elemental composition that confirmed the biosynthesis of pure *Cu NPs*. As a potential utility of the biosynthesized *Cu NPs* as nano adsorbents to the removal of the Cefixime (*Xim*) from the pharmaceutical wastewater. The as-prepared *Cu NPs* may be used as a platform for a drug delivery system and microelectronic chips, heat transfer tools, and water remediation activity, especially in *Xim* from pharmaceutical wastewater. Antibacterial activity of the *Xim* and *Xim@Cu NPs* was conducted assuring the high capability to kill the microorganism, especially Gram-positive bacteria.

## Data Availability

All data generated or analyzed during this study are included in this article and the raw data is available from the corresponding author if it requested.
